# Dataset for effects of the transition from dry forest to pasture on diversity and structure of bacterial communities in Northeastern Brazil

**DOI:** 10.1016/j.dib.2022.107842

**Published:** 2022-01-19

**Authors:** Diogo Paes da Costa, Ademir Sérgio Ferreira Araujo, Arthur Prudêncio de Araujo Pereira, Lucas William Mendes, Rafaela Felix da França, Thallyta das Graças Espíndola da Silva, Julyana Braga de Oliveira, Jenifer Sthephanie Araujo, Gustavo Pereira Duda, Rômulo Simões Cezar Menezes, Erika Valente de Medeiros

**Affiliations:** a Microbiology and Enzimology Lab., Federal University of Agreste Pernambuco, 55292-270 Garanhuns, PE, Brazil; b Soil Quality Lab., Agricultural Science Center, Federal University of Piauí, 64049-550 Teresina, PI, Brazil; c Microbial Ecology and Biotechnology Lab., Federal University of Ceará, 60020-181 Fortaleza, CE, Brazil; d Center for Nuclear Energy in Agriculture, University of Sao Paulo, 13400-970 Piracicaba, SP, Brazil; e Department of Soils, Federal Rural University of Rio de Janeiro, 23890-000 Seropédica, RJ, Brazil; f Department of Nuclear Energy, Federal University of Pernambuco, 50740-540 Recife, PE, Brazil

**Keywords:** Caatinga biome, Tropical soil, Microbial ecology, 16S rRNA, NDVI

## Abstract

The data included in this article supplement the research article titled “Forest-to-pasture conversion modifies the soil bacterial community in Brazilian dry forest Caatinga (manuscript ID: STOTEN-D-21-19067R1)”. This data article included the analysis of 18 chemical variables in 36 composite samples (included 4 replicates) of soils from the Microregion of Garanhuns (Northeast Brazil) and also partial 16S rRNA gene sequences from genomic DNA extracted from 27 of these samples (included 3 best quality replicates) for paired-end sequencing (up to 2 × 300 bp) in Illumina MiSeq platform (NCBI - BioProject accession: PRJNA753707). Soils were collected in August 2018 in a tropical subhumid region from the Brazilian Caatinga, along with 27 composite samples from the aboveground part of pastures to determine nutritional quality based on leaf N content. The analysis of variance (ANOVA) and post-hoc tests of environmental data and the main alpha-diversity indices based on linear mixed models (LMM) were represented in the tables. In this case, the collection region (C1 – Brejão, C2 – Garanhuns, and C3 – São João) was the random-effect variable and adjacent habitats formed by a forest (FO) and two pastures (PA and PB succeeded by this forest) composed the fixed-effect variable (land cover), both nested within C. In addition, a table with similarity percentages breakdown (SIMPER) was also shown, a procedure to assess the average percent contribution of individual phyla and bacterial classes. The figures showed the details of the study location, sampling procedure, vegetation status through the Normalized Difference Vegetation Index (NDVI), in addition to the general abundance and composition of the main bacterial phyla.

## Specifications Table

**Table:** 

Subject	Environmental Science: Environmental Genomics and Metagenomics Earth and Planetary Sciences: Geographical Information System Biological sciences: Bioinformatics
Specific subject area	Application of Bioinformatics based on data from Geographical Information System and genomic sequencing to infer impacts on soil bacterial communities
Type of data	Figures Tables
How the data were acquired	Chemical analyses were done to characterize fertility and enzyme activity in soils of the State of Pernambuco, Brazil. Genetic data were obtained from 16S rRNA gene libraries constructed from 27 soil genomic DNA samples, keeping triplicates of the best quality samples. The library was prepared for paired-end sequencing (up to 2 × 300 bp) using the Illumina MiSeq platform and the raw data were retrieved in FASTA format. Multispectral and Panchromatic Wide-Scan Camera (WPM) images (CBERS-04A satellite) were obtained from the INPE database.
Data format	Raw Analyzed
Description of data collection	Soils were collected in August 2018 in a tropical subhumid region from the Brazilian Caatinga. In all, 36 composite soil samples from the 0-10 cm bed were collected (12 forests and 24 pasture). The aerial part of the pastures was also collected for analysis of nitrogen content.
Data source location	The raw data were obtained from collections carried out in pastures and forests located in three cities in the State of Pernambuco, Northeast Brazil: Brejão (8°59′39.95″S; 36°32′22.69″W), Garanhuns (8°58′27.25″S; 36°27′8.12″W) and São João (8°48′35.77″S; 36°24′25.19″W).
Data accessibility	Repository name: Mendeley Data (V4) Data identification number (DOI number): 10.17632/483vh8mdrv.4 Direct link to the dataset: https://data.mendeley.com/datasets/483vh8mdrv/4
Related research article	Costa D.P., Araujo, A.S.F., Pereira, A.PA., Mendes, L.W., França, R.F., Silva, T.G.E., Oliveira, J.B., Araujo, J.S., Duda, G.P., Menezes, R.S.C., Medeiros, E.V., 2022. Forest-to-pasture conversion modifies the soil bacterial community in Brazilian dry forest Caatinga. Science of the Total Environment. 810, 151943. https://doi.org/10.1016/j.scitotenv.2021.151943

## Value of the Data

• “The dataset provides relevant information about the main effects of conversion from native dry forest to pasture on chemical and biological variables of the soils, especially on enzyme activity and on the structure, composition, and diversity of bacterial communities in the first 10 cm of soil depth.”
• “Researchers interested in bioinformatics, soil fertility, environmental conservation, microbial ecology, and remote sensing aimed at pasture recovery and monitoring of successional forest areas will find this dataset valuable.”
• “The data can be used to study changes in bacterial community structure due to changes in land cover and land use in semi-arid regions. In addition, the data can be used in metagenomic predictions based on the 16S rRNA gene, ecological model building, and various Bioinformatics applications.”

## 1 Data Description

**Fig. 1:**
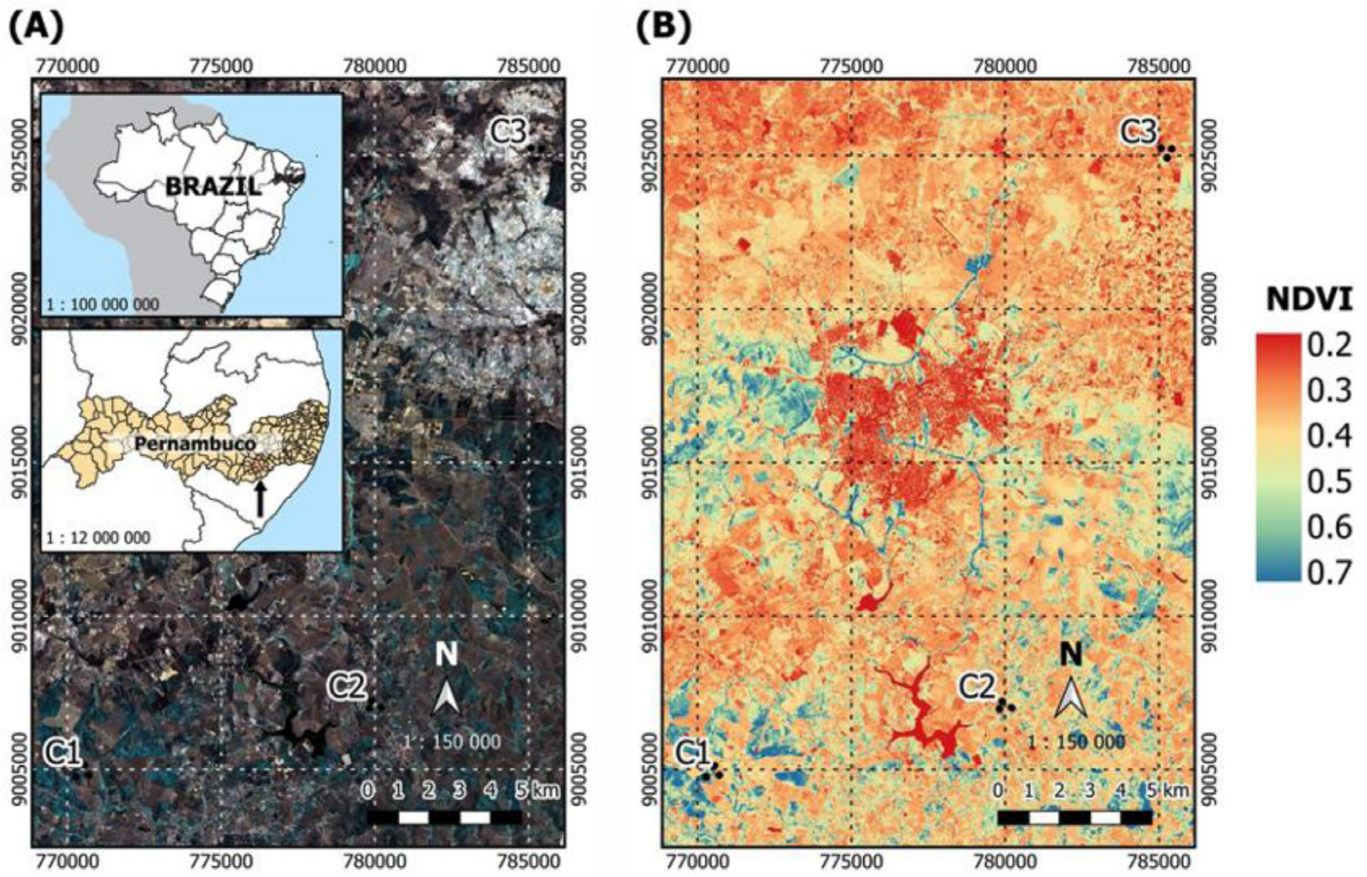
**Spatial characterization, soil cover and location of collection zones in Garanhuns-Region. (A)** This figure demonstrates the location of the state of Pernambuco in Brazil. The image in background corresponds to the fusion of the 3/4/2 RGB color composition bands photographed by CBERS-04A satellite, showing C1 (Brejão: 8°59′39.95″S; 36°32′22.69″W), C2 (Garanhuns: 8°58′27.25″S; 36°27′8.12″W), and C3 (São João: 8°48′35.77″S; 36°24′25.19″W) collection regions. **(B)** The NDVI map of the correspondent area. Ranges from -1 to 1, corresponding to the lowest and highest possible theoretical photosynthetic rate, respectively.

**Fig. 2:**
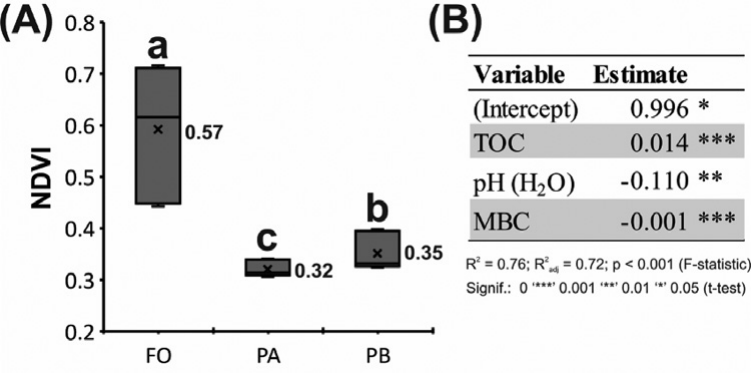
**Dispersions of Normalized Difference Vegetation index (NDVI) average of pasture A (PA), pasture B (PB) and forest (FO). (A)** All niches differed from each other by CL for the estimated marginal means (‘x’ in box-plot) with Bonferroni correction at the p = 0.05 significance level. **(B)** Linear model of NDVI as a function of soil variables. The coefficients for the reduced model with predictive variables of significant influence (t-test, p < 0.05) were estimated, maintaining the same variance of full model, according to the Permutational Multivariate Analysis of Variance - PERMANOVA (p < 0.05). In this case, pH and the dynamics between TOC and MBC in soil were the most important variables in explaining the NDVI oscillation.

**Fig. 3:**
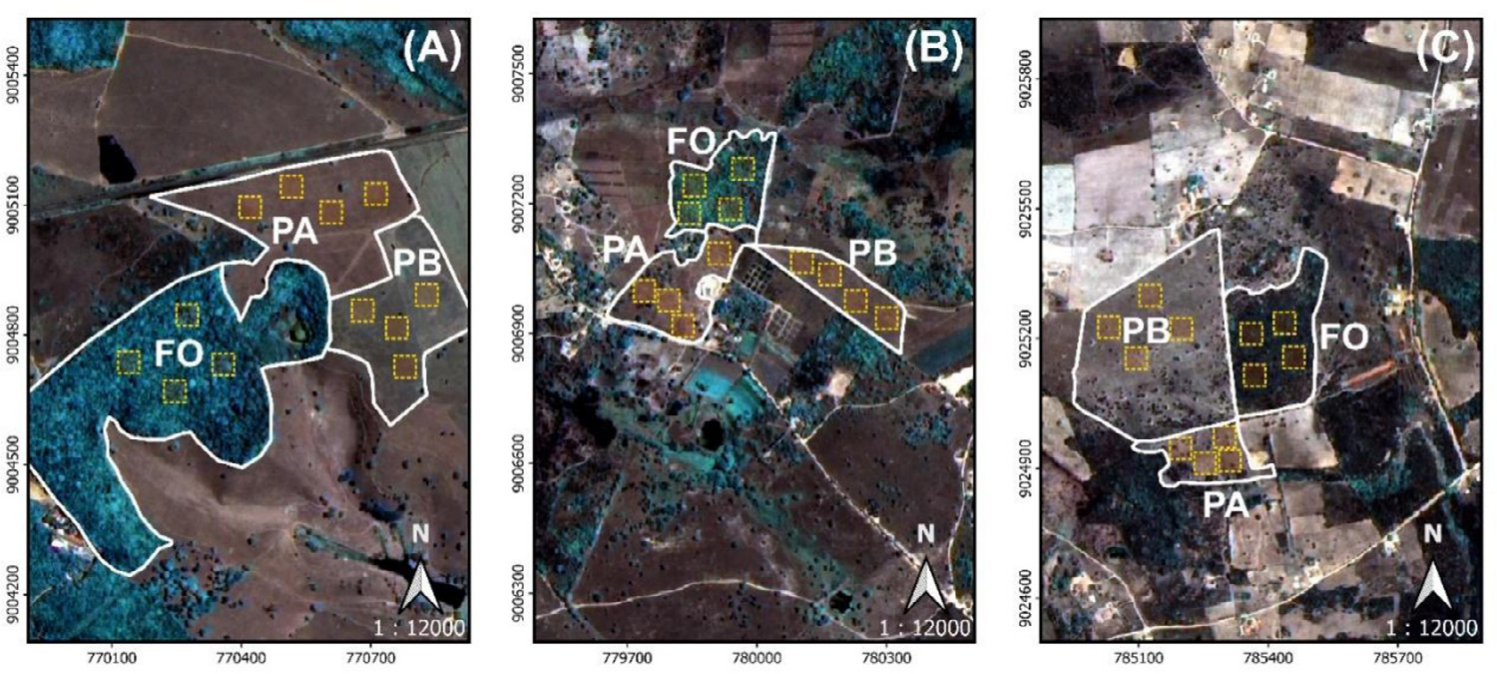
**Spatial detail of collection zones C1 (A), C2 (B) and C3 (C) and locations (outlines in white) of the respective habitats of forest (FO), most active pasture (BP) and least active pasture (PA), according to NDVI index and total leaf nitrogen content in pastures.** Yellow dashed quadrants are the compositional areas of each sample (2.5 ha). The images correspond to the fusion of the 3/4/2 RGB color composition bands photographed by WPM instrument of the CBERS-04A satellite with 2 m spatial resolution (INPE – Brazil). The coordinate reference system was SIRGAS 2000 / UTM zone 24S (EPSG:4674).

The raw data deposited include the panchromatic image at 2 m resolution and the multispectral compositions of the study area at 8 m resolution. Both had the same frame, photographed by the Wide-Scan Camera (WPM) sensor of the CBERS-04A satellite with radiometric and geometric system corrections refined by the use of control points and a digital elevation model (level 4 processing). The imaged swath of this frame was 92 km, indicating a raw data rate of 1800.8 Mbps in the panchromatic image and 450.2 Mbps in the spectral images. The spectral bands provided were: PAN - Panchromatic (B0: 0.45-0.90 µm); B - Blue (B1: 0.45-0.52 µm); G - Green (B2: 0.52-0.59 µm); R - Red (B3: 0.63-0.69 µm); and NIR - Near Infrared (B4: 0.77-0.89 µm). These data were used to study and choose the collection areas (Fig. 1A), to calculate the Normalized Difference Vegetation Index (NDVI) for pastures and forests (Fig. 1B), to differentiate the productivity levels of the studied pastures (Fig. 2A), to detect the most influential variables on NDVI through linear models (Fig. 2B), in the statistical design and sampling procedures (Fig. 3). Under these conditions, 36 composite soil samples were equally distributed (12 samples) among three habitats: forest (FO), less productive pastures (PA), and more productive pastures (PB), according to NDVI values; both nested in three distinct cities (Fig. 3), constituting 3 habitats x 3 cities x 4 replicates.

In addition to the remote sensing data, the raw data related to the chemical variables of the 36 sampled soils were available, being: pH, available P, Ca^2+^, Mg^2+^, Na^+^, K^+^, Al^3+^, potential acidity (H+Al), electrical conductivity (EC), total organic carbon (TOC), organic matter (OM), microbial biomass carbon (MBC), base sum (SB), base saturation (V%), alumina saturation (m%), total cation exchange capacity (TCEC) and effective cation exchange capacity (ECEC), and the activity of the enzymes alkaline phosphatase (EC 3. 1.3.3.1), acid phosphatase (EC 3.1.3.2), β-glucosidase (EC 3.2.1.21) and urease (EC 3.5.1.5). In addition, the mean leaf nitrogen values of the 24 pastures are available. Statistics to identify the assumptions of heterogeneity, normality, and analysis of variance (ANOVA) of these variables (Table 1.a) and Post-hoc tests (Table 2.a) for significant differences between forest (FO) and the two pasture patterns (PA and PB) considered in this study, according to Linear Mixed-effects Models (LMM), were also demonstrated.

**Table 1:** ANOVA table with tests of fixed-effect (niches) and random-effect (collects) terms in the Linear Mixed-effects Models (LMM) for soil properties, leaf nitrogen content in pastures, NDVI, and Alpha diversity measures.

			ANOVA (LMMs)					
	Model contrasts		Random		Fixed		Normality	
	LRT	p (χ^2^)	F	p	F	p	SW	p
**a. Soil and vegetation variables**								
pH in H_2_O	0.6	0.423	4640.7	**<.001**	26.1	**<.001**	0.954	0.142
pH in CaCl_2_	14.9	**<.001**	648.9	**<.001**	31.5	**<.001**	0.942	0.060
P	21.0	**<.001**	60.9	**0.010**	6.4	**0.005**	0.949	0.095
Ca^2+^	24.9	**<.001**	0.3	0.625	0.1	0.913	0.983	0.835
Mg^2+^	62.2	**<.001**	0.2	0.699	1.2	0.323	0.966	0.367
Na^+^	18.6	**<.001**	125.4	**0.004**	2.7	0.082	0.950	0.116
K^+^	0.5	0.488	394.1	**<.001**	1.2	0.303	0.953	0.143
Al^3+^	0.0	0.959	14.2	**0.002**	10.0	**<.001**	0.942	0.060
H^+^+Al^3+^	1.7	0.194	551.4	**<.001**	17.3	**<.001**	0.937	0.050
TCEC	41.3	**<.001**	78.9	**0.010**	2.7	0.082	0.962	0.268
V (%)	36.4	**<.001**	79.2	**0.010**	1.9	0.163	0.961	0.233
TOC	64.2	**<.001**	25.1	**0.036**	26.4	**<.001**	0.971	0.455
MBC	12.6	**<.001**	83.0	**0.005**	5.3	**0.010**	0.981	0.803
EC	29.3	**<.001**	662.2	**0.001**	7.0	**0.003**	0.973	0.516
Aci.P	0.4	0.513	734.6	**<.001**	0.0	0.951	0.967	0.346
Alk.P	0.5	0.471	3791.0	**<.001**	24.5	**<.001**	0.982	0.819
Beta	13.6	**<.001**	567.7	**<.001**	0.9	0.412	0.980	0.732
Ure	12.5	**<.001**	98.7	**0.003**	0.2	0.810	0.962	0.241
LN	15.1	**<.001**	660.2	**0.001**	26.4	**<.001**	0.942	0.181
NDVI	14.6	**<.001**	57.6	**0.008**	119.5	**<.001**	0.929	0.074

**b. Alpha diversity measures**								
Observed	4.2	**0.039**	11672.7	**<.001**	1.1	0.358	0.982	0.899
Shannon	8.5	**0.004**	25784.8	**<.001**	4.2	**0.028**	0.977	0.806
Simpson	16.8	**<.001**	30.4	**0.020**	5.6	**0.011**	0.939	0.113
Fisher	21.1	**<.001**	232.9	**0.002**	7.6	**0.003**	0.959	0.355
Pileous	4.2	**0.040**	4312.4	**<.001**	1.1	0.360	0.982	0.897

(a) Values of REML-likelihood ratio tests (LRT) compared two hierarchically nested models to determine whether the random-effect was significant (p < 0.05);

(b) Deviance analysis for linear models indicated the F values and respective probability tests, according to ANOVA for both fixed and random effects;

(c) Dispersion of residuals was analyzed by the Shapiro-Wilk test (SW), where p > 0.05 confirms the assumptions of normality of LMM. Significant p-values (< 0.05) are highlighted in bold.

**Table 2:** Post-hoc test to soil properties, leaf nitrogen content in pastures, NDVI, and alpha diversity measures.

			Confidence Limits (CL - 95%)								
	Error		Forest			Pasture A			Pasture B		
	SE	(df)	EMM	lower	upper	EMM	lower	upper	EMM	lower	upper
**a. Soil and vegetation variables**											
pH (H_2_O)	0.02	(7.22)	1.63	1.55	1.70	1.79	1.71	1.86	1.82	1.75	1.90
pH (CaCl_2_)	0.06	(2.56)	1.43	1.12	1.75	1.61	1.29	1.93	1.69	1.37	2.01
P	0.27	(2.38)	2.11	0.46	3.77	2.38	0.72	4.03	2.59	0.94	4.25
Ca^2+^	0.63	(2.3)	0.35	−3.65	4.36	0.35	−3.66	4.35	0.45	−3.55	4.46
Mg^2+^	1.02	(2.05)	0.45	−7.05	7.96	0.39	−7.11	7.89	0.17	−7.37	7.70
Na^+^	0.24	(2.4)	−2.67	−4.08	−1.26	−2.84	−4.28	−1.40	−2.96	−4.40	−1.52
K^+^	0.07	(8.4)	−1.43	−1.65	−1.21	−1.55	−1.77	−1.33	−1.43	−1.65	−1.21
Al^3+^	0.22	(13.6)	−0.83	−1.42	−0.23	−1.70	−2.30	−1.10	−2.18	−2.78	−1.59
H^+^+Al^3+^	0.08	(4.98)	2.03	1.75	2.32	1.65	1.36	1.93	1.50	1.21	1.79
TCEC	0.27	(2.13)	2.40	0.53	4.27	2.36	0.47	4.25	2.21	0.32	4.10
V (%)	0.38	(2.17)	3.42	0.79	6.04	3.61	0.99	6.23	3.67	1.05	6.29
TOC	0.47	(2.06)	2.35	−1.10	5.79	1.76	−1.68	5.20	1.72	−1.72	5.16
MBC	0.41	(2.75)	3.68	1.48	5.88	4.15	1.95	6.35	3.30	1.16	5.44
EC	0.22	(2.24)	5.57	4.15	6.99	5.27	3.85	6.69	5.53	4.11	6.96
Aci.P	0.17	(8.05)	4.72	4.20	5.25	4.76	4.23	5.28	4.69	4.16	5.21
Alk.P	0.08	(7.65)	5.20	4.95	5.46	4.65	4.39	4.90	4.52	4.26	4.78
Beta	0.17	(2.62)	4.01	3.08	4.93	4.12	3.20	5.04	3.99	3.07	4.92
Ure	0.37	(2.68)	3.63	1.66	5.59	3.68	1.72	5.65	3.53	1.57	5.50
LN	0.15	(2.22)	—	—	—	3.91	3.08	4.75	4.27	3.43	5.10
NDVI	0.07	(2.44)	−0.55	−0.81	−0.30	−1.14	−1.40	−0.87	−1.05	−1.31	−0.79

**b. Alpha diversity measures**											
Observed	0.06	(3.67)	6.75	6.49	7.02	6.75	6.49	7.01	6.82	6.56	7.08
Shannon	0.01	(3.03)	1.84	1.78	1.90	1.83	1.77	1.88	1.85	1.79	1.91
Simpson	0.00	(2.42)	0.00	0.00	0.00	0.00	0.00	0.00	0.00	0.00	0.00
Fisher	0.00	(2.31)	−0.07	−0.10	−0.04	−0.08	−0.11	−0.05	−0.07	−0.10	−0.04
Pileous	0.08	(3.68)	5.22	4.89	5.55	5.21	4.88	5.54	5.30	4.97	5.64

(a) Variance analysis table show F-values, respective p-values, standard error (SE) values of the difference, and degree of freedom (df) for fixed-effects in each LMM;

(b) Habitats with intervals that do not overlap are significantly different by confidence limits (CL) for the estimated marginal means (EMMs) with Bonferroni correction at the p = 0.05 significance level.

**Fig. 4:**
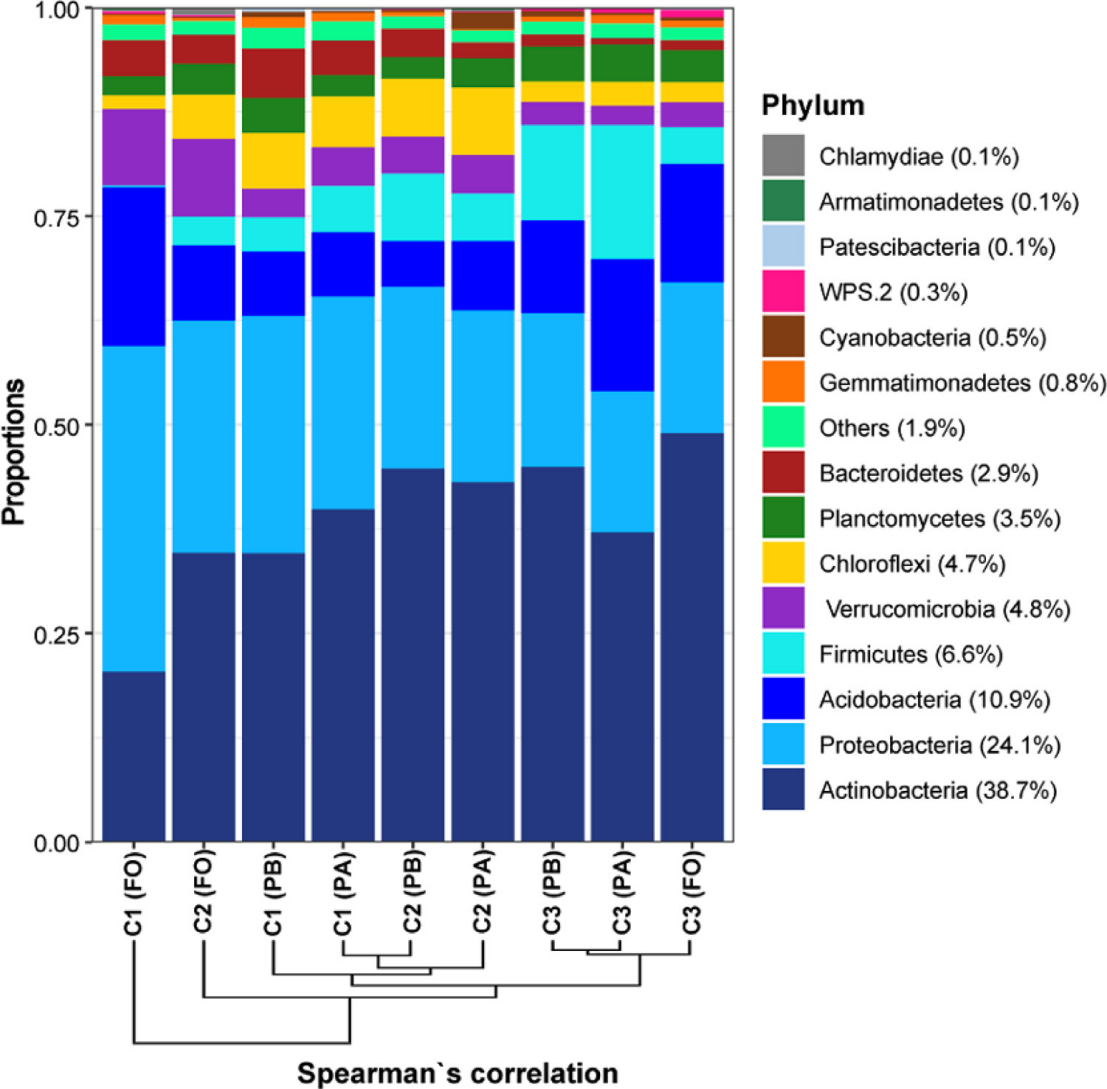
**Structure and relative abundance profile of the main bacterial phyla in soils of pasture A (PA), pasture B (PB) and forests (FO) in the three sampled cities (C1 – Brejão, C2 – Garanhuns, and C3 – São João).** The phyla were arranged in ascending order of abundance from the bottom to the top, and the collections were distanced according to the Spearman's-ρ coefficient for 15 grouped ranks.

The genetic sequences available were from 27 of the 36 total samples, representing the samples with higher concentration and quality of the purified genomic DNA after extraction in soil, evaluated in a NanoDrop® 2000 spectrophotometer (Thermo Fisher Scientific Inc., Waltham, MA, USA). These samples were properly identified in the file "Soil Chemistry and Enzymes.xlsx", available at the Mendley Data link. These sequences consist of 16S rRNA libraries amplified with the primers Bakt_341F and Bakt_805R [1] on the Illumina MiSeq platform (paired-end: 2 × 300 bp). These data were trimmed, filtered, the ends (reverse and forward) were paired-end and chimeric sequences were removed. The structure, composition, and relative abundance of the main bacterial phyla detected in the nine distinct environments were presented (Fig. 4), as well as the statistics of the assumptions (Table 1.b) and of the significant differences in α-diversity indices between forest and the two grasslands (Table 2.b), also according to LMM. In addition, ANOVA and post-hoc test were done for the relative abundance data of the phyla (Table 3) and contribution of major phyla and classes to the dissimilarity between FO, PA, and PB was also calculated to weigh the participation of their respective components in these niches (Table 4).

## 2 Experimental Design, Materials and Methods

### 2.1 Experimental design

The study was conducted in August 2018 in a tropical subhumid region from the Pernambuco state, Northeastern Brazil. Soil bacterial communities and soil variables was assessed using a sampling design based on a linear mixed model (LMM), where the sampling geographic region (C1 – Brejão, C2 – Garanhuns, and C3 – São João) was the random-effect variable (secondary factor) and habitats formed by a forest (FO) and two pastures (PA and PB) composed the fixed-effect variable (land cover as the main factor), both nested within geographic region. The study of independent variables via LMM is considered a weighted approach for biological systems because it demonstrates the overall response of fixed effects (land cover) nested within the random effect (geographic regions), where the latter absorbs variation in the intercepts of the statistical model [2]. Four 2.5 ha quadrats (replicates) were randomly located at each of the nine sampling sites (3 cities x 3 habitats), totalling 36 composite soil samples for chemical and genetic analyses and 24 composite pasture aerial samples for foliar nitrogen determination.

**Table 3:** ANOVA table with significance tests of fixed-effect (niches) in the LMM on the abundance of each bacterial phylum found.

	ANOVA (fixed):		Post-hoc test:		Transformed (yt)						Raw % (y)		
Phylum	F	p (F)	SE	(df)	FO		PA		PB		FO	PA	PB
Actinobacteria	2.30	0.126	2.54	(3.37)	35.74		39.19		40.00		34.6	40.0	41.4
Proteobacteria	9.80	**0.001**	2.62	(2.26)	31.89	**a**	27.16	**b**	28.48	**b**	28.3	21.0	22.9
Acidobacteria	8.60	**0.002**	1.84	(2.90)	21.80	**a**	18.66	**ab**	16.35	**b**	14.1	10.6	8.1
Firmicutes	19.90	**0.000**	2.81	(2.41)	8.39	**b**	16.84	**a**	15.78	**a**	2.7	9.1	7.9
Verrucomicrobia	17.70	**0.000**	1.56	(2.40)	15.07	**a**	11.17	**b**	10.75	**b**	7.1	3.9	3.5
Chloroflexi	10.00	**0.001**	1.76	(2.40)	9.82	**b**	13.51	**a**	12.99	**a**	3.1	5.7	5.3
Planctomycetes	0.30	0.745	0.73	(4.63)	10.35		10.67		10.93		3.3	3.5	3.7
Bacteroidetes	3.60	**0.043**	2.00	(2.30)	9.43	**ab**	8.02	**b**	10.43	**a**	3.0	2.2	3.6
Others	0.90	0.418	0.40	(4.48)	6.70		7.24		7.04		1.4	1.6	1.5
Gemmatimonadetes	0.50	0.608	0.87	(2.60)	4.79		4.44		4.96		0.8	0.7	0.8
Cyanobacteria	2.80	0.082	0.65	(11.80)	2.58		4.67		3.87		0.2	0.9	0.5
WPS-2	24.20	**0.000**	0.97	(2.19)	3.72	**a**	1.92	**b**	1.40	**b**	0.5	0.2	0.1
Patescibacteria	0.60	0.576	0.49	(4.85)	1.90		1.93		2.39		0.1	0.2	0.2
Armatimonadetes	2.40	0.116	0.41	(13.20)	1.91		1.94		0.83		0.2	0.2	
Chlamydiae	1.90	0.175	0.58	(7.62)	1.91		0.67		0.73		0.3	<.1	<.1
Elusimicrobia	1.40	0.278	0.26	(13.20)	1.88		1.30		1.78		0.1	0.1	0.1
Nitrospirae	11.80	**0.000**	0.45	(2.55)	<.01	**b**	0.49	**b**	1.27	**a**	<.1	<.1	0.1
Spirochaetes	0.50	0.643	0.34	(13.20)	0.49		0.68		0.23		0.1	<.1	<.2
FCPU426	14.60	**0.000**	0.32	(3.34)	1.31	**a**	0.15	**b**	<.01	**b**	0.1	<.1	<.1
Fibrobacteres	2.80	0.082	0.21	(13.20)	0.43		0.46		1.07		<.1	<.1	<.1
Rokubacteria	2.30	0.121	0.36	(3.16)	0.13		0.70		0.60		<.1	<.1	<.1
Dependentiae	0.40	0.672	0.23	(10.26)	0.67		0.44		0.70		<.1	<.1	<.1
BRC1	1.40	0.264	0.13	(13.20)	<.01		0.26		0.27		<.1	<.1	<.1
WS2	1.40	0.274	0.14	(4.32)	<.01		0.11		0.23		<.1	<.1	<.1
WS4	2.20	0.135	0.11	(6.78)	<.01		0.24		<.01		<.1	<.1	<.1
Tenericutes	0.60	0.575	0.08	(13.20)	0.07		<.01		0.11		<.1	<.1	<.1
FBP	0.50	0.607	0.06	(13.20)	0.07		0.08		<.01		<.1	<.1	<.1
Epsilonbacteraeota	1.00	0.384	0.05	(13.20)	<.01		<.01		0.08		<.1	<.1	<.1
Omnitrophicaeota	1.00	0.384	0.04	(13.20)	0.07		<.01		<.01		<.1	<.1	<.1
TOTAL											100	100	100

(a) ANOVA done with the raw % data (y%) transformed by the function (yt) = sin^−1^[√(y%⁄100)] 180/π;

(b) Variance analysis table show F-values, respective probability tests, standard error (SE) values of the difference, and degree of freedom (df) for fixed-effects in each LMM;

(c) Post-hoc contrasts followed by the same letter between columns are equal by confidence limits (CL) for the estimated marginal means (EMMs) with Bonferroni correction at the p = 0.05 significance level;

(d) Significant p-values (< 0.05) are highlighted in bold and at the end are the original percentages.

**Table 4:** Contributions of the main phyla and classes of bacteria (%) to the dissimilarity (AD) between the three environments.

				Mean abundance %		
Taxon	AD	Contribuition %	Cumulative %	FO	PA	PB
**a. Phylum**						
Actinobacteria	5.24	25.52	25.52	34.62	40.00	41.42
Proteobacteria	4.06	19.78	45.30	28.36	20.99	22.90
Firmicutes	2.96	14.42	59.72	2.69	9.12	7.88
Acidobacteria	2.77	13.49	73.21	14.08	10.63	8.09
Verrucomicrobia	1.59	7.76	80.97	7.14	3.85	3.55
Chloroflexi	1.46	7.10	88.06	3.11	5.68	5.32
Bacteroidetes	1.11	5.39	93.45	2.99	2.25	3.58
Planctomycetes	0.61	2.97	96.41	3.28	3.52	3.69
Others	0.48	2.33	98.74	2.98	3.27	2.79
Gemmatimonadetes	0.26	1.26	100.00	0.75	0.70	0.80

**b. Class**						
Acidobacteriia	3.77	14.22	14.22	12.83	7.29	4.80
Actinobacteria	2.92	11.04	25.26	17.26	16.57	18.45
Bacilli	2.92	11.03	36.29	2.50	8.81	7.61
Thermoleophilia	2.80	10.59	46.88	13.82	19.33	18.55
Alphaproteobacteria	2.15	8.11	54.99	17.93	13.75	13.03
Gammaproteobacteria	1.77	6.69	61.68	6.41	3.87	5.89
Verrucomicrobiae	1.59	6.02	67.70	7.14	3.85	3.54
Bacteroidia	1.10	4.15	71.84	2.90	2.19	3.50
Deltaproteobacteria	0.94	3.57	75.41	3.87	3.37	3.91
Others	0.81	3.06	78.47	4.79	4.98	5.00
KD4-96	0.76	2.86	81.33	0.23	1.25	1.98
Blastocatellia (Subgroup 4)	0.69	2.60	83.93	0.30	1.59	1.47
Ktedonobacteria	0.68	2.58	86.52	1.56	1.80	0.92
Phycisphaerae	0.52	1.96	88.47	1.99	2.29	2.40
TK10	0.51	1.94	90.41	0.52	1.54	1.01
Acidimicrobiia	0.49	1.87	92.28	2.50	2.77	3.09
Subgroup 6	0.43	1.64	93.91	0.13	0.66	1.02
Oxyphotobacteria	0.32	1.19	95.10	0.09	0.78	0.46
Planctomycetacia	0.26	0.99	96.09	1.17	1.12	1.11
MB-A2-108	0.24	0.91	97.00	0.14	0.49	0.48
Gemmatimonadetes	0.23	0.88	97.88	0.70	0.63	0.73
Chloroflexia	0.16	0.60	98.48	0.17	0.30	0.53
Subgroup 5	0.15	0.58	99.06	0.49	0.27	0.17
AD3	0.14	0.54	99.60	0.46	0.17	0.16
Holophagae	0.11	0.40	100.00	0.12	0.32	0.20

(a) The overall average dissimilarity to phylum was equal to 20.5 according to the Bray-Curtis index.

(b) The overall average dissimilarity to class was equal to 26.5 according to the Bray-Curtis index.

### 2.2 Sample collection

Each of the 36 soil samples or the 27 pastures were composed of 10 subsamples randomly collected in each quadrant to ensure the principle of homogeneity. The pasture samples were cut 10 cm above the surface and the soil samples were collected from the 0 to 10 cm layer, added to plastic bags, and preserved on site in thermal boxes with ice. Then, the samples were taken to Microbiology and Enzymology Laboratory of the Federal University of Agreste Pernambuco (Garanhuns - PE, Brazil), where part of the soils were separated and preserved in ultra-freezer at -80°C for further chemical and enzymatic analysis and genomic DNA extraction.

### 2.3 Analytical approaches

The physicochemical properties of the soils were determined according to the methodologies provided in the EMBRAPA manual [3], verifying soil texture, pH in water (1:2.5 v:v), pH in CaCl_2_ (1:2.5 v:v), Al, H+Al, P, Ca, K, Mg, and Na content. The methodologies for determining the total organic carbon (TOC), microbial biomass carbon (MBC) and for quantifying the activities of the enzymes β-glucosidase (Beta), acid phosphatase (Aci.P), alkaline phosphatase (Alk.P), and urease (Ure) in the soils have been detailed in the main research article related to this data article.

### 2.4 Map editing and NDVI calculation

The maps of the studied region were edited based on the panchromatic and multispectral images from the WPM sensor of the CBERS-04A satellite (L4) made available on the INPE website (http://www.dgi.inpe.br). The images were processed using QGIS 3.10.3 software (http://www.qgis.org), using the coordinate system SIRGAS 2000 / UTM zone 24S (EPSG:4674). Merging of the RGB bands was done to assess vegetation cover and suitability of the areas for collection. Then, Atmospheric correction of the images was performed using the Semi-Automatic Classification Plugin version 7.0.0.1 [4] and the red (B3: 0.63 - 0.69 µm) and near infrared (B4: 0.77 - 0.89 µm) spectral bands were used to calculate the NDVI = (NIR-R) / (NIR+R).

### 2.5 Determination of foliar nitrogen in pasture

The leaf nitrogen was estimated by adapting the sulfur digestion method of Malavolta et al. [5]. The digest solution was prepared in a 1000 mL beaker by adding the substances in the following order: 175 mL of distilled water, 3.6 g Na_2_SeO_3_, 21.39 g Na_2_SO_4_, 4.0 g CuSO_4_ 5H_2_O and finally 200 mL of concentrated H_2_SO_4_. The ground samples of plant material (sieved on 2 mm mesh) were weighed (100 mg) and digested in tubes with 7 mL of the digesting solution, raising the temperature of the digester block by 50°C every 30 minutes until it reached 350°C, remaining at this temperature until the solution became colorless or slightly greenish. Next, the digestion tubes were attached to the nitrogen distiller and slowly added with 18 mol L^−1^ NaOH solution until the coloration turned greenish-brown. At the distiller outlet, a conical flask was positioned with 10 mL of the boric acid indicator solution [20 g boric acid (H_3_BO_3_); 1000 mL of distilled water; 15 mL of 0.1% alcoholic solution of Bromocresol Green (C_21_H_14_Br_4_O_5_S); and 6 mL of 0.1% alcoholic solution of Methyl Red (C_15_H_15_N_3_O_2_)], continuing the process until the solution volume was doubled and the color became slightly greenish. After digestion, the solution was titrated with H_2_SO_4_ 0.02 mol L^−1^ until the indicator turned from green to blue. The volume spent was noted in mL (V), and the percentage of nitrogen in the substrate (%N) was calculated using the expression: %N = 0.28V [5].

### 2.6 Sequences processing

A total of 1,997,557 raw sequence pairs (forward and reverse) read by Illumna MiSeq sequencing were analyzed using the `DADA2' pipeline version 1.16 [6] in R version 3.6.3 [7] in conjunction with RStudio 1.4.1717 [8]. The FIGARO tools [9] were used to optimize the truncation length parameters by ``filterAndTrim'' R function (276 bases for forward reads and 209 bases for reverse reads). According to this tool, forward and reverse reads with higher than 4 and 3 expected errors (maxEE) were discarded, respectively. Next, the error rates of the sequences were calculated with the ``learnErrors'' function, a machine learning-based algorithm; the amplicon sequence variants (ASVs) were inferred using the ``given'' function; and the paired reads were merged by applying the outputs of the previous functions to the input of ``mergePairs''. Chimeric sequences were identified using the ``removeBimeraDenovo'' function and then taxonomic assignments were given the remaining sequences based on the Silva SSU 132 (modified) database [10], using the ``IdTaxa'' algorithm from the `DECIPHER' v 2.20 R library [11], considered a method with classification performance that is better than the standard set by the naive Bayesian classifier [12].

### 2.7 Statistical analysis

Statistical analyses were also done in R version 3.6.3 [7] in conjunction with RStudio 1.4.1717 [8]. The natural log (ln) transformation was used in the raw data to ensure that the data pertained to a normal distribution with constant variance, adding a small adjustment (0.001) on all observations to eliminate errors with the ln transformation before the analysis of variance and checking the assumptions of normality and heteroscedasticity. Variables expressed as percentages (y%) were transformed by the function sin^−1^[√(y%⁄100)]180/π. These transformations are recommended to control error rates in biological data, generating yielded acceptable residual analyses versus fit plots and show p-values similar to the originals data [13]. Analyses were conducted either by Linear Mixed-effects Models (LMM) fitted using the `lmer' function from the `statistics' R package [7] and the algorithms of the `lme4' R package [14]. The ANALysis Of SIMilarity (ANOSIM) test was used to calculate the contribution of phyla and classes to dissimilarity in each habitat (forest and grassland) using Past 4.0 software [15]. Analysis of deviance was done using ANOVA type III Wald F tests with Kenward-Roger degree of freedom (df) for both fixed and random effects. All chemical and enzymatic analyses used 36 composite soil samples [3 cities x 3 niches (FO, PA, and PB) x 4 repetitions]. Pasture nitrogen contents were determined on 24 leaf samples [3 cities x 2 pastures (PA and PB) x 4 repetitions]. Statistics of molecular data from genome sequencing were performed on 27 samples [3 cities x 3 niches (FO, PA, and PB) x 3 repetitions], as only the three best quality genomic DNA samples were sequenced.

## Ethics Statements

There is no ethical issue for this study as no animals or patients were involved in data acquisition.

## CRediT Author Statement

**D.P.C.:** conceptualization, designed the experimental, performed the collection of samples and analytical approaches, performed the data analysis and graphic art, wrote and revised the manuscript; **A.S.F.A.:** analyzed data, wrote and revised the manuscript; **A.P.A.P.:** analyzed data, wrote and revised the manuscript; **L.W.M.:** analyzed data, wrote and revised the manuscript; **R.F.F.:** performed the analytical approaches; **T.G.E.S.:** performed the collection of samples and analytical approaches; **J.B.O.:** performed the collection of samples and analytical approaches; **J.S.A.:** performed the collection of samples and analytical approaches; **G.P.D.:** provided technical and financial support; **R.S.C.M.:** funding acquisition; **E.V.M.:** funding acquisition, wrote and revised the manuscript.

## Declaration of Competing Interest

The authors declare that they have no known competing financial interests or personal relationships that could have appeared to influence the work reported in this paper.
